# A New Dental College

**Published:** 1879-07

**Authors:** 


### A NEW DENTAL COLLEGE.

A Dental Department has just been organized in connection with, and under the auspices of Vanderbilt University, at Nashville, Tenn., with the following organization, viz.:

Dr. W. H. Morgan, Dean; Dr. J. C. Ross, President of the Faculty; Dr. R. R. Freeman, Secretary. The Faculty consists of Dr. W. H. Morgan, Professor of Clinical Dentistry and Dental Pathology; Dr. J. C. Ross, Professor of Operative Dentistry and Dental Hygiene; Dr. R. R. Freeman, Professor of Mechanical and Corrective Dentistry; Dr. J. R. Buist, Professor of Oral Surgery and Surgical Pathology; Dr. T. A. Atchinson, Professor of Materia Medica and Special Therapeutics; Dr. D. R. Stubblefield, Professor of Anatomy and Physiology; Dr. R. W. Steger, Professor of Chemistry and Microscopy; Dr. N. T. Lupton, Emeritus Professor of Chemistry and Metallurgy; Dr. Henry W. Morgan, Assistant to the Chair of Operative Dentistry; Dr. A. S. Duval, Demonstrator of Operative Dentisty; Dr. J. H. Webber, Demonstrator of Mechanical Dentistry; Dr. J. A. Sanduskey, Assistant Demonstrator of Operative Dentistry; Dr. R. B. Lees, Assistant Demonstrator of Mechanical Dentistry.

The resources that Vanderbilt University can afford, and doubtless will bestow, added to and supporting the organization, as here presented, it is difficult to see how anything short of a grand success can result.

Such names as Morgan, Ross and Freeman make a tower of strength for the enterprise. It has been said, "There are already too many dental colleges." If no higher ground is to be taken than some have occupied hitherto, the statement is probably true. But colleges established under such circumstances as this can put the standard high and maintain it, and it is much to be desired that this be done in this instance. If this is done it will meet the approval and support of the profession. But if it begins, from expediency, at a low level; with the promise to work up, then real success will be less certain.

